# Ileal interposition surgery for treatment of type 2 diabetes mellitus-pros and cons

**DOI:** 10.1186/s40200-015-0202-x

**Published:** 2015-10-07

**Authors:** M. Payab, Sh. Hasani-Ranjbar

**Affiliations:** Obesity and Eating Habits Research Center, Endocrinology and Metabolism Molecular -Cellular Sciences Institute, Tehran University of Medical Sciences, Tehran, Iran; Endocrinology and Metabolism Research Center, Endocrinology and Metabolism Clinical Sciences Institute, Tehran University of Medical Sciences, Tehran, Iran

## Abstract

Nowadays, the surgical techniques for treating type 2 diabetes (T2DM) include: Ileal Interposition (II), Ileal Interposition in combination with Sleeve Gastrectomy (IISG) and Diverted Sleeve Gastrectomy (IIDSG). These procedures are not only for obese subjects, but are also used for non-obese subjects. These types of surgical procedures can improve glycemic control, and lead to a significant reduction in oral hypoglycemic agents (OHAs) and insulin therapy. The results of various studies have shown the safety, feasibility, and efficacy of the surgical procedure for the treatment of T2DM. Although it is an effective treatment option, this procedure is not recommended for general use and long-term studies are needed to confirm these findings and potential side effects on a larger number of patients.

## Background

Obesity, type 2 diabetes (T2DM) and metabolic disorders are the most major health problems with an increasing prevalence globally. The two latter complications are directly related to the obesity, while some other complications are results of metabolic abnormality associated with obesity [1, 2].

Diabetes mellitus is a chronic disease and a common cause of morbidity and mortality around the world. The global prevalence of diabetes among adults (aged 20–79 years) in 2010 was 6.4 % (285 million adults) [3]. Also, world Health Organization (WHO) has reported that by 2030, the prevalence of T2DM will increase to 336 million subjects [4]. Considering the global health crisis of diabetes and obesity in this era and the need for an effective treatment of patients, surgery can be proposed in addition to drug therapy (Synthetic and Medicinal plants) [5, 6].

In the past, other surgical procedures including sleeve gastrectomy (SG), gastric banding, Roux-en-Y-Gastric Bypass (RYGB) and Jejuno-Ileal Bypass (JIB) were used to treat diabetes. But today, other surgical procedures have been modified to achieve the maximum remission in diabetic patients [7, 8]. Now, the surgical techniques for treating type 2 diabetes include: Ileal Interposition (II), Ileal Interposition in combination with Sleeve Gastrectomy (IISG) and Diverted Sleeve Gastrectomy (IIDSG) [7, 9]. These procedures are not limited to remission of T2DM, beside hypertension, hyperlipidemia, obesity, and obstructive sleep apnea may also be influenced [10–13].

## Main text

Ileal Interposition is not only specifically a bariatric surgery, but also is used to treat diseases that target the metabolic syndrome such as T2DM. Moreover, both foregut (excluding of the duodenum) and hindgut mechanisms are utilized in these procedures [11, 14, 15]. These procedures not only for obese subjects, but is also used for non-obese subjects [8, 16–18].

Several studies have shown that these types of surgical procedures can improve glycemic control, (including reductions in HbA1c, fasting blood glucose (FBG), postprandial glucose (PPG)) and also may improve the metabolic parameters. These results persisted in all patients during the 3-year follow-up [12, 16–18].

Weight loss by these procedures was attributed to the increased release of peptides secreted in the ileum, GLP-1 and peptide YY (PYY), which has anorectic effects [19]. In the ileal interposition procedure, earlier exposure of food to ileum and rapid stimulation of the interposed ileal segment by ingested food leads to augmented glucagon-like peptide 1 (GLP-1) secretion [12, 17]. GLP-1 resulted in delaying gastric emptying, promotes satiety, suppressing appetite, inhibiting glucagon secretion, decreasing gluconeogenesis, and stimulating the glycogenesis [20, 21].

Ghrelin stimulates the secretion of hyperglycemic hormones such as glucagon, cortisol and growth hormone and also inhibit insulin secretion. Ghrelin is an orexigenic hormone (appetite stimulant). In the SG component of the IISG and IIDSG procedures, serum level of ghrelin is reduced and patients feel satiety and restrict their caloric intake [22–24].

Ileal Interposition is a procedure that does not lead to malabsorption. In IIDSG procedure, duodenum and part of the jejunum are bypassed and can cause malabsorption. All patients are recommended to take iron, calcium, B12, and multivitamins supplementation regularly [12, 25].

De paula et al. in their study have reported objective improvement of retinopathy and symptomatic improvement in neuropathy [18]. Also, other studies have reported that after the metabolic surgery, a significant reduction in oral hypoglycemic agents (OHAs) and insulin therapy was observed for glycemic control [11, 17, 18].

Usually, patients with shorter duration of diabetes, higher BMI (Body Mass Index) and higher C-peptide level respond better to these surgical procedures [11, 26, 27].

In some studies minor and early postoperative complications such as vomiting, esophagitis, bowel obstruction, gout, and urinary tract infection have been observed. But so far, no major long-term surgical complications have been reported [18, 23]. In advanced T2DM, iatrogenic hypoglycemia can be a limiting factor for these procedures [28].

In a study published recently, Ugale et al. introduced a novel tool known as Diabetes Remission Score (DRS) for choosing the type of surgery and predicting diabetes remission following IISG or IIDSG. The study aimed at presenting an effective and useful method based on parameters such as the duration of diabetes, BMI and stimulated C-peptide response prior to surgery for predicting post surgery diabetes remission [8]. The DRS included three grades as grade 1 (mild, DRS 7–8), grade 2 (moderate, 9–11) and grade 3 (severe, DRS 12–14). Higher scores could be associated with lower chance of remission.

In conclusion, the results of various studies have shown the safety, feasibility, and efficacy of the surgical procedure for the treatment of T2DM. Although it is an effective treatment option, this procedure is not recommended for general use and long-term studies are needed to confirm these findings and potential side effects on a larger number of patients.

## Abbreviations

T2DM: Type 2 diabetes mellitus
II: Ileal Interposition
IISG: Ileal Interposition Sleeve Gastrectomy
IIDSG: Ileal Interposition Diverted Sleeve Gastrectomy
FBG: Fasting blood glucose
PPG: Post prandial glucose
GLP-1: Glucagon-like peptide 1
BMI: Body mass index
